# Neutralization of Tier-2 Viruses and Epitope Profiling of Plasma Antibodies from Human Immunodeficiency Virus Type 1 Infected Donors from India

**DOI:** 10.1371/journal.pone.0043704

**Published:** 2012-08-31

**Authors:** Raiees Andrabi, Manju Bala, Rajesh Kumar, Naveet Wig, Anjali Hazarika, Kalpana Luthra

**Affiliations:** 1 Department of Biochemistry, C. N. Centre, All India Institute of Medical Sciences, New Delhi, India; 2 Department of Medicine, C. N. Centre, All India Institute of Medical Sciences, New Delhi, India; 3 Blood Bank, C. N. Centre, All India Institute of Medical Sciences, New Delhi, India; 4 Regional STD Teaching Training & Research Centre, Safdarjang Hospital, New Delhi, India; University of South Carolina School of Medicine, United States ofAmerica

## Abstract

Broadly cross neutralizing antibodies (NAbs) are generated in a group of HIV-1 infected individuals during the natural infection, but little is known about their prevalence in patients infected with viral subtypes from different geographical regions. We tested here the neutralizing efficiency of plasma antibodies from 80 HIV-1 infected antiretroviral drug naive patients against a panel of subtype-B and C tier 2 viruses. We detected cross-neutralizing antibodies in approximately 19–27% of the plasma, however the subtype-C specific neutralization efficiency predominated (p = 0.004). The neutralizing activity was shown to be exclusively mediated by the immunoglobulin G (IgG) fraction in the representative plasma samples. Epitope mapping of three, the most cross-neutralizing plasma (CNP) AIIMS206, AIIMS239 and AIIMS249 with consensus-C overlapping envelope peptides revealed ten different binding specificities with only V3 and IDR being common. The V3 and IDR were highly antigenic regions but no correlation between their reciprocal Max50 binding titers and neutralization was observed. In addition, the neutralizing activity of CNP was not substantially reduced by V3 and gp41 peptides except a modest contribution of MPER peptide. The MPER was rarely recognized by plasma antibodies though antibody depletion and competition experiments demonstrated MPER dependent neutralization in two out of three CNP. Interestingly, the binding specificity of one of the CNP (AIIMS206) overlapped with broadly neutralizing mAb 2F5 epitope. Overall, the data suggest that, despite the low immunogenicity of HIV-1 MPER, the antibodies directed to this region may serve as crucial reagents for HIV-1 vaccine design.

## Introduction

The development of an immunogen capable of eliciting neutralizing antibody (NAb) response against HIV-1 remains elusive primarily due to enormous viral diversity [1]–[3]. The progress is hampered in part by inadequate information about the mechanisms associated with complex immune responses evoked during the natural course of HIV-1 infection [4]–[7]. Even the most promising antibody based vaccine candidates have shown effectiveness against limited number of HIV-1 strains [8]–[11]. However, results from a recent HIV-1 vaccine trial (RV144) in Thailand demonstrated for the first time, a vaccine induced protection in humans [12]. Although the study of immune correlates in the recipients of RV144 vaccine revealed that only high titer of plasma anti-V1/V2 antibodies correlated with lower risk of HIV infection, the induction of broadly cross-neutralizing antibodies still remains a main goal for the development of HIV vaccine [13]. Indeed, studies have shown that broad and potent NAb responses develop in the sera of a subset of HIV-1 infected individuals, and dissecting the nature of these responses may provide important clues for the design of new vaccine immunogens [14]–[17]. Analysis of the antibody response in HIV-1 infected individuals have revealed their specificities to all the HIV-1 proteins but antibodies directed mainly to the envelope glycoproteins (gp120 and gp41) are capable of mediating virus neutralization [16]–[18].

The non-covalently associated HIV-1 envelope glycoproteins, which mediate receptor binding and viral entry into the host cells [19], [20], remain the sole viral targets for neutralizing antibodies [16], [21], [22]. The CD4 binding site (CD4bs) and co-receptor binding region (third variable (V3) loop) of gp120 have been shown to serve as major vulnerable targets for HIV-1 neutralization [15], [16], [21], [23]–[27], although the role of other regions is also documented [28]–[31]. In comparison, the gp41 is highly conserved and is divided into three major regions, the extracellular region, the transmembrane (TM) domain and the cytoplasmic tail (CT) [32]. Antibody reactivity has been observed to be associated with different regions of the gp41 in HIV-1 infected donors, including the N and C-heptad regions [33], [34], immunodominant loop (ID) [35], [36], membrane proximal external region (MPER) [37]–[39] and C-terminal (CT) [40] of which the MPER constitutes the major target for broadly neutralizing antibodies on HIV-1 gp41 [22], [39].

Subtype-C remains the major HIV-1 infecting clade accounting for approximately 50% infections worldwide, with its primary centre being Africa followed by India [1]. Majority of the knowledge, as it relates to HIV-1 neutralizing antibody responses in subtype-C viruses, primarily come from African patients [41]–[44] with limited information on Indian subtype-C viruses despite the considerable differences between the viruses from these two geographical regions [45]–[48].

The study was aimed to examine the percentage of cross neutralizing plasma antibodies and identification of major linear antigenic regions in subtype-C HIV-1 infected Indian patients which has not yet been addressed in a great detail. Overall, our data suggest that, a good percentage of cross neutralizing antibodies circulate in these patients and the antibodies directed to the MPER region of HIV-1 gp41 envelope protein may serve as an important neutralization epitope.

## Materials and Methods

### Ethics Statement

The study was approved by the ethics committee of All India Institute of Medical Sciences (AIIMS) and the written informed consent was obtained from all the participants.

### Plasma samples

Eighty HIV-1 seropositive patients enrolled in this study were recruited from the Regional STD Teaching Training & Research centre, Safdarjang hospital, and department of Medicine All India Institute of Medical Sciences (AIIMS), New Delhi, India. The patient samples were collected between the year 2008 and 2010. The HIV-1 seronegative samples were collected from the Blood bank, C.N. Centre, AIIMS, New Delhi. All the patients were tested and diagnosed hepatitis and tuberculosis negative. The whole blood samples of HIV-1 positive donors were collected in EDTA vacutainers, plasma was separated by centrifugation at 300 g and stored in aliquots at −80°C until use. The plasma samples were heat inactivated at 56°C for 1 h before using in the assays.

### Monoclonal antibodies, peptides and recombinant proteins

The monoclonal antibodies (mAbs) used in this study were 447-52D, 1418 kindly provided by S. Zolla Pazner, and 2F5 and 4E10 (contributed by H. Katinger), were obtained through NIH AIDS Research and Reference Reagent Program (NIH, ARRRP). The 6 consensus subtype-B (con-B) MPER linear overlapping peptides and a complete set of 212 linear peptides (each 15 mer with an 11 amino acid overlap or a 4 amino acid walk) corresponding to the complete sequence of consensus subtype-C (con-C) gp120 and gp41 envelope proteins were obtained from the NIH, ARRRP. Three peptides corresponding to the con-C V3 (35 mer) (CTRPNNNTRKSIRIGPGQTFYATGDIIGDIRQAHC), 25 mer MPER (DLLALDSWKNLWNWFDITNWLWYIK) and 19 mer immunodominant region (IDR) (LGIWGCSGKLICTTAVPWN) were synthesized from Sigma Genosys, USA. The peptides had >95% purity. Three recombinant gp120s representing sequences of primary HIV-1 isolates from subtype-A, B and C (produced in 293 cells) were purchased from Immune Technology Corp. (New York, NY).

### Determination of plasma viral load, CD4 count and total IgG levels

The plasma viral load was determined commercially (Lifeline Laboratories, New Delhi) using a real time PCR based method. The viral load was represented as RNA copies/ml of plasma. The CD4 counts were determined by flow cytometry. Total IgG content of the plasma was assessed by using a commercial ELISA kit from Ray Biotech, Inc USA. The clinical and demographic data are summarized in Table S1.

### Purification of the polyclonal immunoglobulin G (IgG) fractions

Three plasma samples, AIIMS206, AIIMS239 and AIIMS249, identified as the most cross-neutralizing in this study, one plasma sample from HIV-1 seronegative healthy donor A1, were used for this experiment. Briefly, the polyclonal IgG was purified from the plasma samples by using protein A affinity chromatography (GE Healthcare) according to the manufacturer's instructions. IgG was eluted from the columns in 0.1 M citric acid, pH 3.0. Column fractions containing IgG were neutralized, pooled, and dialyzed against phosphate-buffered saline (PBS), pH 7.4. IgG purity was determined by sodium dodecyl sulfate-polyacrylamide gel electrophoresis, and the concentration was determined by measuring the relative absorbance at 280 nm and also by non-commercial total IgG quantitation ELISA.

### Antibody binding assay (ELISA)

The test peptides (V3, MPER, IDR and con-C gp160 overlapping peptides) were immobilized on 96-well high binding ELISA plates (Corning) by an overnight incubation at 4°C of 100 µl of peptide per well diluted at 1 µg/ml concentration in 50 mM bicarbonate buffer (pH 9.6) except the MPER peptide which was adjusted equimolar to the V3 peptide concentration. Unbound peptides were removed by washing thrice with phosphate-buffered saline (PBS) containing 0.2% Tween 20 (Sigma-Aldrich) (PBST), and the plates were blocked with 200 µl of Roswell Park Memorial Institute media (RPMI) containing 15% fetal calf serum (FCS; Hyclone) and 2% bovine serum albumin (BSA; Sigma-Aldrich), incubated for 1 h at 37°C. Next, the plates were washed again three times and 100 µl of each plasma sample at different dilutions were added and incubated for 1.5 h at 37°C. Following 3 washings with PBST, the plates were further incubated for 1.5 h at 37°C with 100 µl (diluted 1/2000 in PBS containing 2% BSA) of alkaline-phosphatase conjugated anti-human IgG Fc (Southern Biotech). Finally the bound antibodies were detected by addition of alkaline phosphatase substrate (Sigma-Aldrich) in 10% diethanolamine buffer, and the colorimetric reaction was stopped by the addition of 6N NaOH. The optical density was read at 405 nm.

The 50% maximal binding of plasma antibodies (Max50) value was calculated for plasma that showed saturation for binding at low plasma dilutions. Initially all the plasma samples were tested at eight dilutions (dilution range: 1/30 to 1/100000) with V3, MPER and IDR but only six dilutions (1/300 to 1/100000) were considered for analysis as the first two dilutions (1/30 and 1/100) could not cross the cutoff for Max50 value which was three times the mean OD405 of the twenty healthy seronegative plasma samples at the lowest dilution plus three standard deviations. For epitope mapping with con-C gp160 overlapping peptides, a similar criterion was employed for Max50 determination with a different dilution range (1/100 to1/3000).

For binding of IgG fraction to con-C and B overlapping MPER peptides, the IgG was concentrated five times to that of original plasma volume that was purified, while for binding to recombinant gp120s the volume was adjusted to equal to that of the original volume of plasma used. For ELISA binding of IgG fractions, the coating concentration of the MPER overlapping peptides was also increased to 4 µg/ml. The binding of the plasma IgG fractions was performed by titrating the purified IgG fractions against MPER peptides and subtype-A, B and C recombinant gp120 proteins. The ELISA binding protocol was similar as above, except that the final reaction with substrate for gp120 ELISA was read in 20 minutes instead of 30 minutes. All the control mAbs were tested with peptides and proteins at a concentration ranging from 10 to 0.00003 µg/ml (12 dilutions). Experiments were carried out in triplicates and repeated at least two times for reproducibility.

### Antibody depletion assay

In order to remove V3, MPER and IDR specific antibodies from the plasma, six passages on V3, MPER and IDR peptide-coated wells were carried out by using the ELISA binding protocol with some modifications described previously [49]. Briefly, we increased the binding capacity by coating the plates with 10 µg/ml peptide concentration. Second, to avoid the deleterious effects of detergent in subsequent neutralization cell cultures, the Tween 20 in the wash solution was replaced by 5% FCS. Third, sequentially, the plasma samples at 1∶30 dilution, were incubated for several successive passages (6 passages) on coated wells to remove the peptide specific antibodies. For this purpose, after 1 h of incubation at 37°C, the plasma were removed by pipetting and further incubated on other coated wells for an additional hour. For controls, the plasma were also mock depleted by serial passages on plates treated in parallel but not coated with peptides. The antibodies removed from the plasma and bound to the peptide-coated wells were then detected by incubation with alkaline phosphatase-conjugated anti-human IgG(Fc) and followed by adding substrate as described above. The diminution in OD405 throughout the successive passages could be monitored and correlated to the sequential depletion of peptide specific antibodies. The depletion percentage in the last passage was calculated as follows: percent depletion = 100−[100×(OD_405_ at the last passage/OD_405_ at the first passage)]. The plasma samples of two healthy HIV-1 seronegative individuals (A1 and A2) were used as ELISA controls for the depletion assay, and minimal upto 5% depletion was observed in the last passage. The depleted fractions of plasma were filtered through 0.45 mm-pore-size filters (Costar) before being tested for their neutralizing activity.

### Primary isolates and pseudoviruses

The two newly generated primary isolates, AIIMS201 and AIIMS212 were isolated in our laboratory by previously described co-cultivation method [50]. Both the viruses belong to subtype-C, based on the partial envelope sequence of gp120 (C2–C5) (Andrabi et al, submitted). The envelope clones of subtype-C Du156.12, ZM109F.PB4, ZM53M.PB12, and subtype-B JRFL, RHPA4259.7, TRO.11 have been previously described [51], [52], and were obtained from the NIH, ARRRP. The pseudotyped viruses were produced by co-transfection of rev/env expression plasmid and an env-deficient HIV-1 backbone vector (pSG3ΔEnv) into exponentially dividing 293T cells in 6-well tissue culture plates (Corning Inc) using calcium phosphate method (Promega Inc). Pseudovirus-containing culture supernatants were harvested 48–53 hours post transfection, filtered (0.45 µm pore size) and stored at −80°C in 1 ml aliquots. The 50% tissue culture infectious dose (TCID50) was determined in TZM-bl cells.

### Neutralization assay

Neutralization was measured as a reduction in luciferase gene expression after a single round of infection of JC53bl-13 cells, also known as TZM-bl cells (ARRRP; catalog no. 8129), with Env-pseudotyped viruses [53]. Briefly, 200 TCID50 of pseudovirus was incubated with heat inactivated diluted plasma samples (dilution range: 60 to 20000) in duplicates in a total volume of 100 µl for 1 hr at 37°C in 96-well flat-bottom culture plates. Freshly trypsinized cells (10,000 cells in 100 µl of growth medium containing 25 µg/ml DEAE Dextran and indianavir (1 µM) in case of primary isolates) were added to each well. One set of control wells received cells plus pseudovirus (virus control) and another set received cells only (background control). After 48 hours of incubation, luciferase activity was measured by using the Bright-Glo Luciferase Assay System (Promega Inc.). Fifty percent infective dose (ID50) values of the plasma were derived by determination of the plasma dilution that neutralized 50% of the infectious virus. Values were calculated through a dose-response curve fit with nonlinear function using GraphPad prism software (San Diego, CA). To compare the neutralization capacity of purified IgG fractions with the corresponding plasma, the IgG fractions for each sample were concentrated to a volume equal to that of the original volume of plasma that was purified.

In peptide interference neutralization assay, a test peptide was preincubated 30 minutes with plasma prior to the addition of virus. The final concentration of peptide in mixtures with plasma and virus was 20 µg/ml. To control for the possible direct effects of peptides on viral infection (i.e., in the absence plasma antibodies), assays were conducted in the presence of peptides (V3, MPER and IDR) at the same concentrations as those used in competition assays. In these experiments, the peptides had either little or no effect on virus infectivity. This internal control level of infection with each peptide was used as a baseline reference for peptide competition assays with plasmas. For competition assay validation, an anti-V3 antibody (447-52D) known to neutralize SF162 (a tier 1 sensitive subtype-B virus) was competed with two separate peptides corresponding to con-C and B V3 sequences (final peptide concentration 10 µg/ml) and tested for neutralization.

### Statistical Analysis

Statistical analyses were performed using Graph Pad Prism 5 for Windows, Graph Pad Software, San Diego, California USA. A non-linear regression curve straight line was plotted using the method of least squares to determine the Max50 and ID50 values. Median reciprocal Max50 binding titers were compared using Wilcoxon matched pairs test or Mann-Whitney U test. Further the Spearman rank test was used to determine the correlation between the two variables. A p-value less than 0.05 was considered significant for this study.

## Results

### Characteristics of HIV-1 infected patients

The details of the 80 HIV-1 infected antiretroviral naïve patients recruited for this study is summarized in Table S1. The patients had been infected for different time periods, ranging from a few days up to seven years (based on time since 1^st^ diagnosis). There were 30 females and 50 males within the age range of 20–57 years. The median viral load determined for 53 patients was 30800 (range = 156–2180000) RNA copies/ml plasma with a few patients having viral load below the detectable limits (<47 copies/ml). The median CD4 count was 337 (range = 14–966) cells/cubic millimeter (n = 80), while the mean plasma total IgG levels was 12.3 mg/ml (n = 65).

### Neutralizing activity of the plasma antibodies from HIV-1 infected patients

In order to determine the neutralizing activity, we tested plasma antibodies against a panel of 5 subtype-C and 3 subtype-B tier 2 viruses (Table 1). Two of the new subtype-C isolates (AIIMS201 and AIIMS212) were tested and displayed resistance to neutralization by broadly neutralizing antibodies (Table S2), and were assigned as tier 2 viruses (Table 1). Overall the viruses were selected based on their subtype, geographical occurrence and resistance to neutralization. Presumably most of the patients were infected with subtype-C which is the major subtype in India [45], [46]. Indeed the subtyping of a few patients from this cohort showed a prominence of subtype-C infections (Andrabi et al, submitted) (Table 2).

**Table 1:** List of HIV-1 viruses used in this study.

Virus	Tier	Subtype	Country of origin	1Acc no.
**AIIMS201**	2	C	India	JF300176
**AIIMS212**	2	C	India	JF300177
**ZM109F.PB4**	2	C	Zambia	AY424138
**Du156.12**	2	C	South Africa	DQ411852.1
**ZM53M.PB12**	2	C	Zambia	AY423984.2
**JRFL**	2	B	USA	U63632
**TRO.11**	2	B	Italy	AY835445.1
**RHPA4259.7**	2	B	USA	AY835447

The viruses were selected based on resistance to neutralization, the clade they belongs to and the geographical origin.

1 Acc no.: Gene bank accession number.

**Table 2:** Neutralization potential of plasma antibodies from HIV-1 patients against subtype-B and C tier 2 viruses.

AIIMS ID	Subtype	^c^AIIMS201	^c^ZM53M.PB12	^c^Du156.12	^b^RHPA4259.7	^b^TRO.11	^c^AIIMS212	^b^JRFL	^c^ZM109F.PB4
**AIIMS206**	**ND**	**>20000**	**17766**	**3016**	**3313**	**4199**	**4022**	**3040**	**3243**
**AIIMS213**	**C**	**1978**	***258***	**1940**	<60	<60	<60	<60	<60
**AIIMS239**	**C**	*783*	**1257**	*317*	*509*	*385*	*457*	*245*	*220*
**AIIMS220**	**ND**	*106*	*480*	**2178**	*796*	*156*	<60	*66*	*84*
**AIIMS914**	**ND**	**2264**	*431*	<60	*119*	*163*	<60	*445*	*113*
**AIIMS905**	**ND**	**2264**	<60	<60	*70*	*269*	<60	*145*	<60
**AIIMS249**	**ND**	*457*	*369*	*189*	*120*	*734*	*520*	*291*	*189*
**AIIMS210**	**C**	**1794**	<60	*102*	<60	<60	<60	*442*	<60
**AIIMS232**	**C**	*210*	*440*	*510*	*214*	<60	*316*	*244*	<60
**AIIMS211**	**ND**	*68*	<60	*222*	**1396**	<60	*124*	<60	<60
**AIIMS225**	**C**	*208*	*90*	**1128**	*96*	<60	*62*	<60	<60
**AIIMS901**	**ND**	*208*	*183*	*291*	<60	*472*	*177*	*308*	<60
**AIIMS287**	**C**	<60	*838*	*302*	<60	<60	<60	*264*	<60
**AIIMS288**	**ND**	<60	*752*	*108*	<60	<60	<60	*76*	<60
**AIIMS904**	**ND**	*618*	*235*	<60	<60	<60	<60	*76*	<60
**AIIMS254**	**C**	*70*	<60	*206*	*446*	*162*	<60	<60	*104*
**AIIMS289**	**ND**	<60	*138*	*352*	*210*	<60	<60	*124*	*88*
**AIIMS212**	**C**	<60	<60	<60	*656*	<60	<60	<60	<60
**AIIMS248**	**ND**	*226*	*88*	*298*	<60	*108*	*88*	<60	<60
**AIIMS284**	**ND**	<60	*334*	*250*	<60	<60	<60	<60	*78*
**AIIMS906**	**ND**	*418*	*70*	*62*	<60	*142*	<60	<60	<60
**AIIMS911**	**ND**	<60	*72*	<60	<60	<60	<60	*463*	<60
**AIIMS909**	**ND**	*405*	<60	<60	<60	*89*	*85*	*75*	<60
**AIIMS250**	**ND**	*110*	*160*	*230*	<60	<60	*84*	<60	<60
**AIIMS291**	**ND**	<60	*292*	*84*	<60	<60	<60	<60	*116*
**AIIMS219**	**ND**	<60	<60	<60	*366*	<60	<60	<60	<60
**AIIMS299**	**ND**	*108*	*74*	<60	*78*	<60	<60	*66*	*264*
**AIIMS234**	**C**	<60	<60	*190*	<60	<60	*182*	<60	<60
**AIIMS255**	**C**	*146*	*90*	<60	<60	<60	*168*	<60	*64*
**AIIMS913**	**ND**	<60	*281*	<60	<60	<60	<60	<60	<60
**AIIMS300**	**ND**	*158*	<60	<60	*112*	<60	<60	<60	*98*
**AIIMS281**	**A**	<60	<60	<60	<60	<60	*98*	*204*	<60
**AIIMS283**	**ND**	<60	*228*	<60	<60	<60	<60	<60	<60
**AIIMS264**	**C**	*78*	<60	*204*	<60	<60	<60	<60	<60
**AIIMS910**	**ND**	*68*	*209*	<60	<60	<60	<60	<60	<60
**AIIMS205**	**ND**	<60	*202*	*66*	<60	<60	<60	<60	<60
**AIIMS242**	**ND**	<60	*170*	*64*	<60	*64*	*82*	<60	<60
**AIIMS274**	**ND**	<60	*74*	*126*	<60	*100*	<60	<60	*78*
**AIIMS277**	**ND**	<60	<60	*80*	*152*	<60	<60	*86*	<60
**AIIMS290**	**C**	<60	*184*	<60	<60	<60	<60	<60	*72*
**AIIMS276**	**ND**	<60	*148*	*82*	<60	<60	<60	*62*	<60
**AIIMS224**	**C**	<60	<60	*160*	<60	<60	<60	<60	<60
**AIIMS208**	**C**	<60	<60	*156*	<60	<60	<60	<60	<60
**AIIMS241**	**C**	*72*	<60	*136*	<60	<60	*64*	<60	<60
**AIIMS253**	**C**	*120*	<60	<60	<60	<60	*88*	<60	<60
**AIIMS297**	**ND**	*80*	*66*	<60	<60	<60	*110*	<60	<60
**AIIMS275**	**C**	<60	<60	*82*	<60	<60	<60	<60	*100*
**AIIMS230**	**ND**	<60	<60	*120*	<60	<60	<60	<60	<60
**AIIMS286**	**ND**	<60	<60	<60	<60	<60	*62*	*110*	<60
**AIIMS273**	**ND**	<60	*76*	<60	*92*	<60	<60	<60	<60
**AIIMS235**	**A**	*82*	<60	<60	<60	<60	*84*	<60	<60
**AIIMS294**	**C**	<60	<60	<60	*70*	<60	<60	*94*	<60
**AIIMS203**	**ND**	<60	<60	*94*	<60	<60	<60	<60	<60
**AIIMS908**	**ND**	<60	<60	<60	<60	*94*	<60	<60	<60
**AIIMS293**	**C**	<60	<60	<60	<60	<60	<60	<60	*86*
**AIIMS231**	**ND**	<60	<60	<60	<60	*74*	*68*	<60	<60
**AIIMS226**	**C**	<60	<60	<60	<60	<60	*78*	<60	<60
**AIIMS272**	**ND**	<60	*76*	<60	<60	<60	<60	<60	<60
**AIIMS216**	**ND**	<60	<60	*70*	<60	<60	<60	<60	<60
**AIIMS285**	**ND**	<60	*70*	<60	<60	<60	<60	<60	<60
**AIIMS271**	**ND**	<60	<60	<60	<60	<60	<60	*68*	<60
**AIIMS298**	**C**	*66*	*62*	<60	<60	<60	<60	<60	<60
**AIIMS233**	**ND**	<60	<60	*66*	<60	<60	<60	<60	<60
**AIIMS915**	**ND**	*65*	*61*	<60	<60	<60	<60	<60	<60
**AIIMS207**	**ND**	<60	<60	<60	<60	<60	<60	<60	<60
**AIIMS218**	**ND**	<60	<60	<60	<60	<60	<60	<60	<60
**AIIMS223**	**ND**	<60	<60	<60	<60	<60	<60	<60	<60
**AIIMS237**	**ND**	<60	<60	<60	<60	<60	<60	<60	<60
**AIIMS244**	**ND**	<60	<60	<60	<60	<60	<60	<60	<60
**AIIMS270**	**ND**	<60	<60	<60	<60	<60	<60	<60	<60
**AIIMS279**	**A**	<60	<60	<60	<60	<60	<60	<60	<60
**AIIMS280**	**ND**	<60	<60	<60	<60	<60	<60	<60	<60
**AIIMS282**	**ND**	<60	<60	<60	<60	<60	<60	<60	<60
**AIIMS292**	**ND**	<60	<60	<60	<60	<60	<60	<60	<60
**AIIMS295**	**C**	<60	<60	<60	<60	<60	<60	<60	<60
**AIIMS296**	**C**	<60	<60	<60	<60	<60	<60	<60	<60
**AIIMS902**	**ND**	<60	<60	<60	<60	<60	<60	<60	<60
**AIIMS903**	**ND**	<60	<60	<60	<60	<60	<60	<60	<60
**AIIMS907**	**ND**	<60	<60	<60	<60	<60	<60	<60	<60
**AIIMS912**	**ND**	<60	<60	<60	<60	<60	<60	<60	<60

The cross neutralizing activities of plasma antibodies from 80 HIV-1 infected drug naïve individuals from north India against the tier 2 subtype-B and C viruses indicated on the top of the table. Each virus is designated with its subtype (subtype-C and subtype-B; superscript alphabet on left). The AIIMS ID of the patient samples is given on the left of the table followed by the subtype of viruses they were infected with (C- subtype-C, A- subtype-A and ND- not determined). The numerical values in boxes are the 50% neutralization titers (ID50) defined as the dilution of plasma which neutralized 50% of viral infection in the assay. For clarity, this information is coded: ID50>1000 (Bold), ID50 = 61–1000 (Italic) and <60, where ID50 was not reached. Each experiment was performed at least two independent times.

Of the 80 plasma samples, 64(80%) were able to neutralize at least one virus while 16(20%) did not show any neutralization. Nevertheless 20(25%) plasma samples were found to neutralize ≥50% viruses tested (Table 2). Only three plasma samples AIIMS206, AIIMS239 and AIIMS249 were able to neutralize all the eight viruses tested, AIIMS206 being the most potent. These three plasma samples are represented as most cross-neutralizing plasma (CNP) of tier 2 viruses tested here. In terms of overall neutralization frequency, 190 of 640 virus/plasma combinations showed neutralizing activity (approximately 29.6%) (Table 2). Among all the viruses, ZM53M.PB12, Du156.12 (subtype-C) were most sensitive while TRO.11 (subtype-B) and ZM109F.PB4 (subtype-C) were most resistant, neutralized by 35(43.7%), 34(42.5%) and 15(18.7%), 16(20%) plasma samples respectively. Although the cross-clade neutralizing activity was observed in most of the neutralizing plasma, however the subtype-specific neutralization predominated, plasma antibodies being more effective against subtype-C than subtype-B viruses (p = 0.004).

### Plasma immunoglobulin-G (IgG) fractions mediate the neutralizing activity

**Figure 1:**
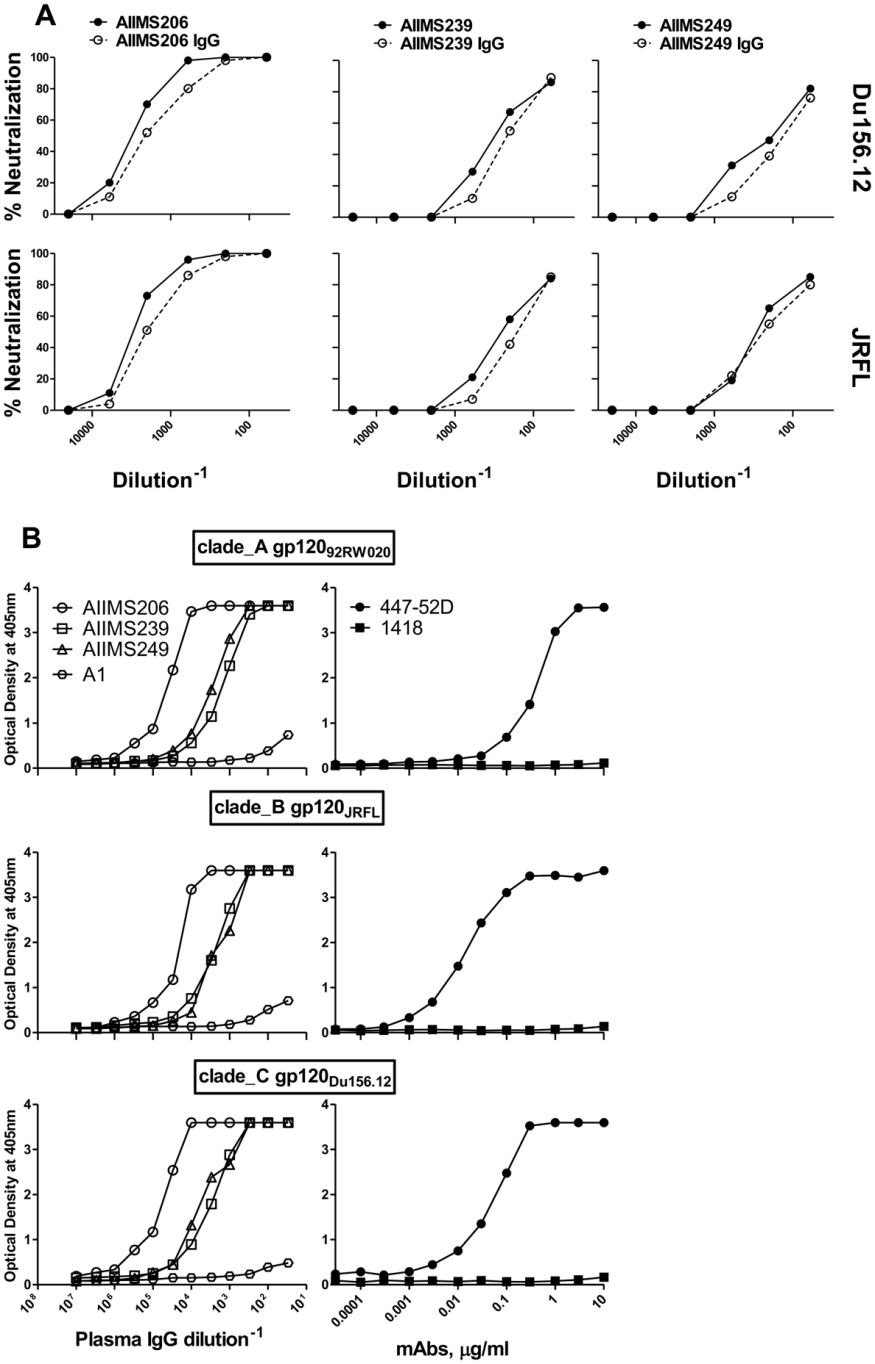
Neutralization and binding of IgG fractions from cross-neutralizing (CNP) HIV-1 plasma. (A) Neutralization curves of IgG fractions purified from CNP on protein-A. The neutralization capacity of purified polyclonal IgG (open circle) is compared with those of the original plasma (solid circle). To allow the comparison, the purified IgG were concentrated to volumes equal to that of the original volume of plasma run over the column. Neutralization of CNP, AIIMS206, AIIMS239 and AIIMS249 column fractions is compared for the subtype C (Du156.12) and subtype B (JRFL) isolates. (B) The binding pattern of IgG fractions of CNP along with IgG from healthy control plasma A1. The ELISA binding was carried out with envelope recombinant gp120 proteins from subtype-A (92RW020), subtype-B (JRFL) and subtype-C (Du156.12) isolates. The anti-V3 (447-52D) and anti-B19 (1418) monoclonal antibodies were used as assay controls.

To determine the component of the HIV-1-infected donor plasma responsible for the neutralizing activity, we purified polyclonal IgG from three CNP, AIIMS206, AIIMS239, AIIMS249 and a healthy seronegative plasma sample A1 on protein A-Sepharose columns. The neutralization capacity of each column fraction was compared to the degree of neutralization in the original plasma. We tested IgG fractions of these plasma with two *env*-pseudotyped viruses from subtype-C (Du156.12) and B (JRFL) in a neutralization assay. In each case, we observed comparable neutralizing activity in the original plasma and purified IgG (Figure 1A). The IgG fractions were also tested for ELISA binding with three recombinant gp120 proteins representing subtype-A (92RW020), B (JRFL) and C (Du156.12) viruses. Consistent with neutralization results, we observed that the IgG fractions from HIV-1 plasma retained cross reactive binding to envelope gp120s (Figure 1B). In addition, we observed little or no binding or neutralization activity in the IgG-depleted fraction (data not shown).

### Epitope mapping of CNP with consensus-C gp160 overlapping peptides

**Figure 2:**
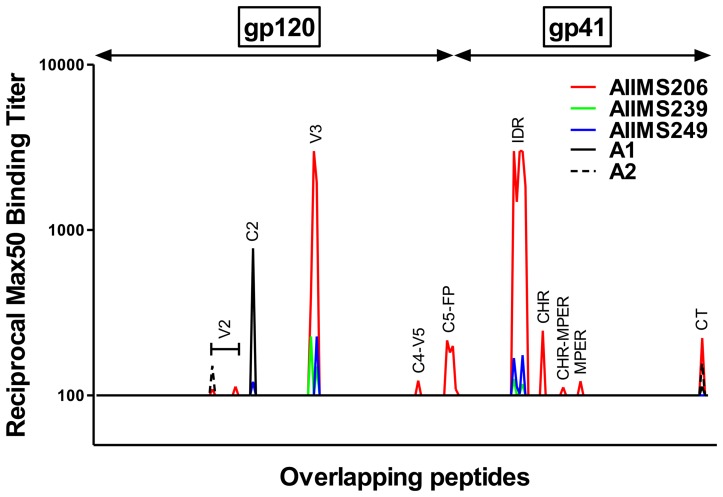
Epitope mapping of polyclonal antibodies from three cross-neutralizing plasma with consensus-C HIV-1 gp160 overlapping peptides. ELISA binding of polyclonal plasma antibodies from three most cross-neutralizing plasma (CNP) AIIMS206, AIIMS239 and AIIMS249 and two healthy seronegative individuals (A1 and A2) to 212 consensus-C HIV-1 envelope glycoprotein gp160 (gp120 and gp41) overlapping linear peptides (15 mer each with 11 amino acid overlap or 4 amino acid walk). The plasma were reacted at 4 dilutions (dilution range: 100 to 3000) and reciprocal Max50 binding titers were calculated using least square regression method. The plasma showed reactivity to second variable (V2), second constant (C2), third variable (V3), fourth constant-fifth variable (C4-V5), fifth constant (C5) regions of gp120 and fusion protein (FP), immunodominant region (IDR), C-heptad region (CHR), membrane proximal external region (MPER) and C-terminal (CT) of gp41 protein.

To identify the role of linear antigenic epitopes on HIV-1 clade C envelope protein in virus neutralization, we tested the reactivity of three CNP samples (AIIMS206, AIIMS239 and AIIMS249) along with two control plasma from healthy seronegative donors with 212 con-C gp160 overlapping peptides in an ELISA binding assay (Table S3a; S3b, Figure 2). We were particularly interested in mapping the antigenic regions common for binding to these CNP. Based on the Max50 ELISA binding titers, the immunoreactivity of CNP mapped to the second variable (V2), second constant (C2), third variable (V3), fourth constant-fifth variable (C4-V5), fifth constant (C5) regions of gp120 and fusion protein (FP), immunodominant region (IDR), C-heptad region (CHR), membrane proximal external region (MPER) and C-terminal (CT) of gp41 protein. Among CNP, AIIMS206 showed highest number of binding specificities (nine), while only two binding sites were shared by all the three CNP (V3 and IDR) (Figure 2).

For further analysis we took MPER in addition to V3 and IDR peptides (common binding specificities for CNP) based on the binding of AIIMS206 and considering that MPER is recognized by broadly neutralizing antibodies [22], [39]. We tested 80 plasma samples for ELISA binding with the con-C V3 (35 mer), IDR (19 mer) and MPER (24 mer) peptides and observed a high percentage of samples reaching Max50 binding titers with V3 (99%), IDR (95%) as compared to MPER (56%). The median Max50 binding titers were also higher with V3 (8706) and IDR (2382) in comparison to MPER (451) (Figure S1). To determine the possible role of anti-V3, anti-IDR and anti-MPER antibodies in neutralization, we compared the Max50 antibody binding titers specific to these three regions with mean ID50 neutralization titers of each plasma sample with all the viruses tested. We did not find any correlation between the neutralization potential and anti-V3 (p = 0.52), anti-IDR (p = 0.59) and anti-MPER (p = 0.97) antibody binding titers (Figure S2). We also did not find any subtype-B or C specific correlation of neutralization with binding. Nonetheless, we observed a modest positive correlation of MPER specific Max50 binding titers with Du156.12 (a subtype-C virus) (p = 0.04) and a negative correlation with JRFL (a subtype-B virus) (p = 0.02) neutralization titers (Figure S2).

### Depletion and competition of V3, IDR and MPER directed plasma antibodies

**Figure 3:**
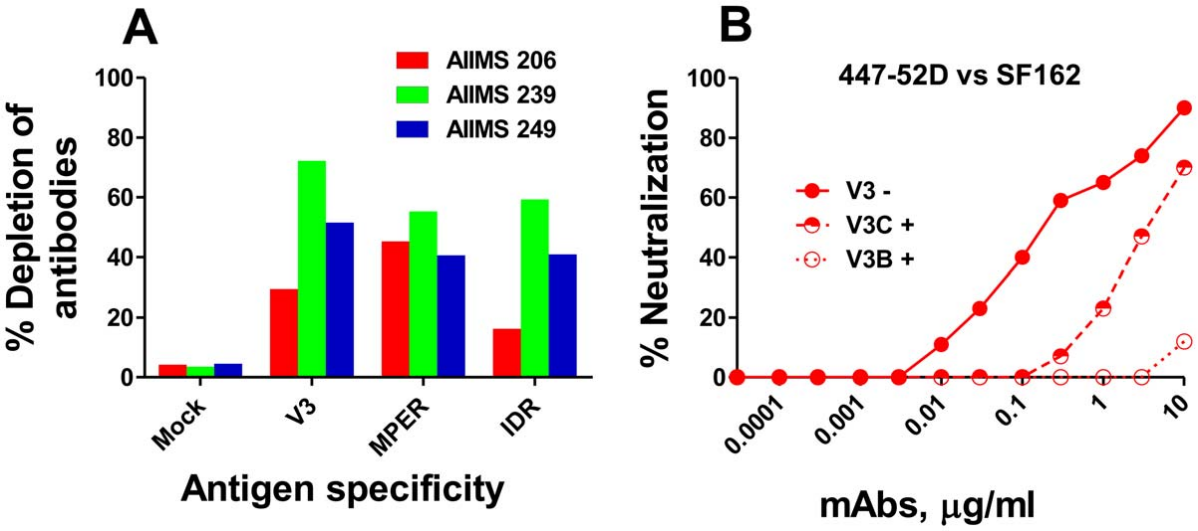
Depletion and competition of plasma antibodies from cross-neutralizing plasma. The depletion and competition of antibodies from three cross-neutralizing plasma (CNP) (AIIMS206, AIIMS239 and AIIMS249) was carried out with V3 (35 mer), MPER (25 mer) and IDR (19 mer) specific peptides. The antibodies from CNP were depleted by passing plasma samples (at 1∶30 dilution) over antigen coated ELISA plates (peptide coating concentration, 10 µg/ml) six times and the percent depletion of antibodies to each region was calculated as: percent depletion = 100−[100×(OD_405_ at the last passage/OD_405_ at the first passage)]. The percentage antibody depletion for CNP against each peptide is shown (A). As control, mock depletion of CNP was carried out on the uncoated plates which showed minimal effect on antibody depletion (A). For competition, CNP were preincubated 30 minutes with the same peptides at a final peptide concentration of 20 µg/ml. Both depleted (designated as ‘D’) and competed (designated as ‘+’) plasma were subsequently tested for neutralization with all eight viruses (data shown in Table 3). To validate the competition assay, we tested 447-52D an anti-V3 antibody known to neutralize SF162 (a tier 1 sensitive subtype-B virus) along with two peptides corresponding to consensus-C and B V3 sequences (at a final concentration, 10 µg/ml) (B).

In order to evaluate the participation of anti-V3, IDR and MPER specific antibodies in viral neutralization, we depleted and competed the plasma antibodies from three CNP mentioned above, with peptides specific to these regions. For antibody depletion, we passed the CNP over antigen coated ELISA plates for six passages and observed a substantial removal (16% to 72%) of antigen specific antibodies (Figure 3A). The mock depleted plasma showed minimal antibody loss. For competition, plasma samples were pre-incubated with peptides at a final peptide concentration of 20 µg/ml. Both depleted ‘D’ and competed ‘+’ plasma samples were subsequently tested for neutralization with all eight viruses (Table 3).

**Table 3:** Effect of V3, MPER and IDR peptides on ID50 neutralization titers of the cross-neutralizing plasma in depletion and competition experiments.

		aID_50_								bID_50_ fold decrease					
Plasma ID	Virus	Untreated plasma	Mock D	V3 D	V3+	MPER D	MPER+	IDR D	ID+	V3 D	V3+	MPER D	MPER+	IDR D	ID+
**AIIMS206**	AIIMS201	>20000	10597	13143	>20000	8362	1370	17776	10891	0.806	1	1.2	**14.5**	0.59	1.83
	AIIMS212	4022	3684	3965	3565	4345	862	3807	3123	0.929	1.12	0.84	**4.66**	0.96	1.28
	Du156.12	3106	2398	2479	2172	2648	1903	2172	2479	0.967	1.43	0.9	1.63	1.1	1.25
	ZM109F.PB4	3243	2737	2737	2924	2829	3123	2280	2924	1	1.1	0.96	1.03	1.2	1.1
	ZM53M.PB12	17766	14259	12495	11697	8982	4641	7129	8670	1.14	1.51	1.58	**3.82**	2	2.04
	JRFL	3040	2648	2829	3123	3123	3448	3456	3564	0.936	0.97	0.84	0.88	0.76	0.85
	RHPA4259.7	3313	2829	1967	1724	2320	1967	2398	2400	1.43	1.92	1.21	1.68	1.17	1.38
	TRO.11	4199	3564	2737	3228	2479	1281	2737	2102	1.3	1.3	1.43	**3.27**	1.3	1.99
**AIIMS239**	AIIMS201	783	543	508	476	390	476	640	731	1.06	1.64	1.39	1.64	0.84	1.07
	AIIMS212	457	331	353	445	281	150	342	431	0.93	1.02	1.17	**3.04**	0.96	1.06
	Du156.12	317	254	230	281	223	101	263	238	1.1	1.12	1.13	**3.1**	0.96	1.33
	ZM109F.PB4	220	230	215	230	108	123	195	246	0.92	0.95	2.12	1.78	1.17	0.89
	ZM53M.PB12	1257	1324	525	620	119	120	921	755	2.52	2.02	**11.12**	**10.47**	1.43	1.66
	JRFL	245	290	281	223	140	78	290	223	1.03	1.09	2.07	**3.14**	1	1.09
	RHPA4259.7	509	445	320	310	171	111	310	246	1.39	1.64	2.6	**4.58**	1.43	2.06
	TRO.11	385	310	286	290	127	104	320	310	1.08	1.32	2.44	**3.7**	0.96	1.24
**AIIMS249**	AIIMS201	457	390	476	662	417	525	419	476	0.819	0.69	0.93	0.87	0.93	0.96
	AIIMS212	520	525	640	492	290	230	640	862	0.82	1.05	1.81	2.26	0.82	0.6
	Du156.12	189	170	166	159	156	106	149	140	1.02	1.18	1.08	1.78	1.14	1.35
	ZM109F.PB4	189	223	177	230	171	119	140	160	1.25	0.82	1.3	1.58	1.59	1.18
	ZM53M.PB12	369	342	342	340	353	202	353	365	1	1.08	0.96	1.82	0.96	1.01
	JRFL	291	281	300	246	246	215	310	271	0.936	1.18	1.14	1.35	0.906	1.07
	RHPA4259.7	120	111	115	115	123	119	111	108	0.965	1.04	0.9	1	1	1.11
	TRO.11	734	755	707	662	755	599	731	720	1.06	1.1	1	1.22	1.03	1.01

The table describes the change in ID50 neutralization titers of the three cross-neutralizing plasma CNP (AIIMS206, AIIMS239 and AIIMS249) against 5 subtype-C and 3 subtype-B viruses upon depletion and competition with V3, MPER and IDR specific peptides.

a **ID_50_**; The numerical values are the ID50 neutralization titers of CNP after depletion (‘D’) (passing plasma over antigen coated ELISA plates @10 µg/ml) and competition ‘+’ (incubating CNP with specific peptides @20 µg/ml) along with untreated and mock (passed CNP on uncoated ELISA plates) controls.

b **ID_50_ fold decrease**; Plasma ID50 fold decrease for depletion assay was calculated as, ID50 with mock depleted plasma/ID50 with V3, MPER and IDR depleted plasma, and for competition assay as ID50 with untreated plasma/ID50 with V3, MPER and IDR treated plasma. The more than threefold change in ID50 neutralization titer was arbitrarily taken as positive. The CNP AIIMS206 and AIIMS239 showed dependence on MPER (Bold) directed antibodies especially with peptide competition while V3 and IDR showed minimal effect on neutralization as compared to untreated and mock plasma controls.

Based on the fact that multiple epitope specificities contribute to overall neutralization of HIV-1, we possibly may not anticipate a complete removal of neutralizing activity by knocking out antibodies directed to a single antibody specificity, however it may slightly alter the potency. Assuming the above for the analysis, we arbitrarily took a threefold change in the ID50 neutralization titer as positive in the depletion and competition experiments. Based on this criteria, the CNP AIIMS206 and AIIMS239 showed dependence on the MPER directed antibodies with four and six viruses respectively, effect being more prominent with peptide competition (Table 3). The V3 and IDR peptides showed minimal effect on the viral neutralization as compared to untreated and mock depleted plasma controls (Table 3). A partial and complete inhibitory effect on neutralization of SF162 by 447-52D was observed with V3-C and V3-B peptides respectively (Figure 3B).

### Epitope mapping of IgG fractions from the CNP with overlapping MPER peptides

**Figure 4:**
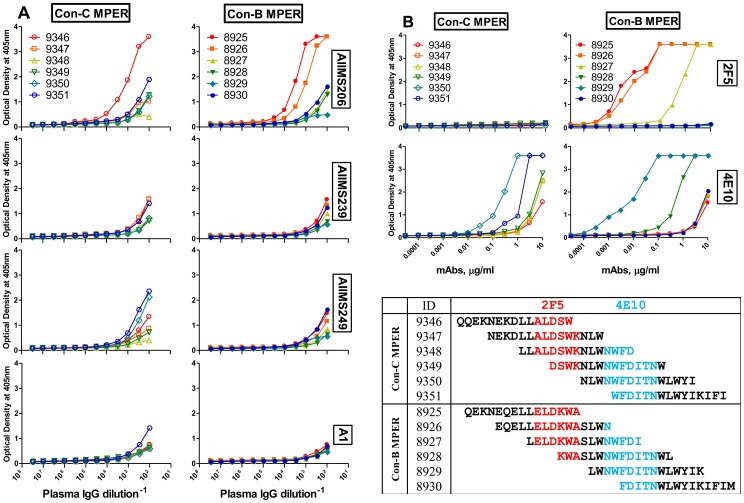
Mapping of IgG fractions with overlapping MPER peptides. (A) ELISA binding curves of IgG fractions from three cross-neutralizing plasma samples, AIIMS206, AIIMS239, AIIMS249 and a healthy seronegative control plasma A1, with linear consensus-C and consensus-B overlapping MPER peptides. The MPER overlapping peptides were coated at 4 µg/ml and the purified IgG fractions were concentrated five times to the volume that of the original plasma (used for IgG purification) and then reacted with peptides at 12 dilutions. (B) Binding pattern of two MPER specific broadly neutralizing mAbs 2F5 and 4E10 with same MPER peptides, which show a specific binding to their respective core epitopes. The 4B (lower) shows a schematic arrangement of consensus-C and B MPER overlapping peptides and the core epitope previously defined for 2F5 (red) and 4E10 (blue) are indicated. Among the CNP, AIIMS206 exhibited strong reactivity with peptides of both subtype-C and B and its binding region was overlapping with 2F5 epitope.

The functional assessment of two out of three CNP, AIIMS206 and AIIMS239 showed MPER antibody directed neutralization dependence with a few viruses, however the precise target epitope within MPER remained to be identified. In this context, we titrated the IgG fractions of the CNP from a higher starting concentration (five times higher than the original plasma), against a set of six each overlapping peptides corresponding to the con-C and B MPER sequences in an ELISA binding assay. The binding curves of CNP show that AIIMS206 exhibited strong reactivity with peptides of both subtype-C and B, while AIIMS239 and AIIMS249 showed no or weak reactivity (Figure 4A). The binding assay was carried out in presence of two MPER specific mAbs, 2F5 and 4E10, known to be broadly neutralizing. The control antibodies 2F5 and 4E10 whose epitopes within MPER have been previously defined [22], showed specific binding to their respective core epitopes (Figure 4B). Interestingly, the reactivity pattern of the CNP, AIIMS206 defined an epitope within MPER, which was overlapping with the mAb 2F5 epitope. However the binding specificity was slightly different as it reacted with subtype-C peptides while 2F5 did not (Figure 4A–B).

## Discussion

Dissecting the specificities of the anti-HIV-1 neutralizing antibodies will assist in identifying the targets for an HIV-1 subunit vaccine. Broadly cross-neutralizing antibodies against HIV-1 are detected in 5–25% of plasma samples and their specificities are different and still analyzed [54]–[56]. We employed here a systematic approach of binding and neutralization to identify the antigenic targets recognized by HIV-1 specific envelope antibodies from the subtype-C infected individuals from India. We found high antigenicity of V3 and IDR as compared to MPER. However, we did not observe any correlation of neutralization with their reciprocal Max50 binding titers, though functional assessment showed a modest MPER dependent neutralization in two out of three cross neutralizing plasma.

The neutralizing activity of the plasma samples was evaluated against a panel of 3 subtype-B and 5 subtype-C tier 2 viruses. We found that 19–44% plasma samples contained neutralizing antibodies depending upon the virus. The neutralizing activity of three representative plasma samples was shown to be mediated by the IgG fractions, consistent with previous reports [57], [58]. Overall the plasma samples exhibited higher neutralizing capability of subtype-C compared to subtype-B viruses, though a substantial proportion of the plasma samples showed cross-clade neutralization potential. A potential reason for this subtype-C specific activity may be due to the limited envelope diversity in infecting viruses which would help greater sharing of the neutralization determinants [59]. Similar results were previously observed in studies conducted on HIV-1 individuals from South Africa [60], [61], Thailand [62], [63], and France [64]. The property of plasma samples showing a better neutralizing capacity against region specific viruses may in part be attributed to the involvement of the plasma antibodies effective against the lineage specific envelope domains in neutralization [47], [65]. Together these data suggest that neutralizing antibody-based vaccine immunogens might face less problems at the regional level to overcome epitope diversity.

This study was also aimed to identify and characterize the major antigenic targets and their relevance with viral neutralization in subtype-C Indian patients. Based on the immunoreactivity of three CNP (AIIMS206, AIIMS239 and AIIMS249) to linear overlapping envelope peptides, the binding specificities mapped to ten different regions on HIV-1 envelope glycoprotein (five each on gp120 and gp41). These regions have also been previously identified in subtype-B and C viruses as potentially important targets for neutralizing antibodies and serotyping [16], [21], [22]. We focussed here on three regions only (V3, IDR, MPER), however the other regions that were less immunodominant could also possibly function as neutralization epitopes and need to be studied in detail. The MPER is highly conserved target of broadly neutralizing mAbs 2F5, 4E10 and Z13, while V3 is a semiconserved target of cross-neutralizing mAb 447-52D [22], [24] and the IDR is not known as a target for bNAbs though highly antigenic [35], [36].

We observed a high immunodominance of V3 and IDR over MPER based on the relative binding titers. The Max50 binding titers for IDR and V3 were approximately 5 to 15 times respectively higher than MPER. Interestingly, a good proportion (56%) of the patient plasma reached Max50 binding value to MPER peptide which is unexpected due to low immunogenicity of this region [66], [67]. However, this high percentage of plasma binding to MPER should not be confused with the presence of 2F5 and 4E10 like bNAbs which are rare in HIV-1 infected individuals [22], [66], [68]. Since the binding was carried out with whole MPER (25mer) peptide in this study, it is possible that we would have scored the non-neutralizing binding antibodies whose epitopes overlap with the epitopes of bNAbs [69], [70].

We were unable to find any statistical correlation of antibody binding titers directed to V3 and IDR with the neutralizing activity, though high antibody titers were recorded in almost all the infected plasma. The functional assessment of these antibodies (V3 and IDR specific), from three CNP showed a little or no effect on neutralization, suggesting that these antibodies possibly do not contribute to neutralization. The findings are in consensus with studies showing that IDR directed antibodies have little role in HIV-1 neutralization [22]. With respect to the V3, a number previous studies carried out in HIV-1 subtype-A, B and C infected individuals have shown a similar effect, wherein despite the binding capability of plasma antibodies to autologous and heterologous V3 peptides, they fail to display any neutralization [29], [57], [60], [71]. The ubiquitous presence of anti-V3 antibodies could be attributed to recognition of “decoy” V3 epitopes exposed on defective monomeric envelope forms, with masking of V3 on the native envelope trimers possibly affecting the accessibility of these antibodies [72]–[75], thereby preventing neutralizing activity.

One of the salient finding of this study was contribution of MPER specific antibodies in neutralization by two CNP, AIIMS206 and AIIMS239. Although we did not find any correlation of neutralization with MPER specific antibody binding titers except a modest positive and negative correlation with Du156.12 and JRFL respectively. However, the ID50 neutralization titers in the functional assays dropped considerably (up to fourteen fold) in two out of three CNP with a few viruses. Incidentally, the effect on viral neutralization was more prominent with competition than antibody depletion though AIIMS239 showed a uniform trend with ZM53M.PB12 (subtype-C) virus. This biased effect could not be explained, however a possible explanation is that inhibition neutralization assays were performed in solution, and therefore, it is likely that all the specific antibodies will be competed better by MPER peptides in solution compared to those adsorbed on the coated plates (in depletion) which might present the MPER peptide in a slightly different conformation. Interestingly, studies have shown that, in solution, linear peptides can efficiently inhibit binding of V3 mAbs to native gp120 proteins [76], [77].

Remarkably, the neutralization dependence on the MPER region in these two plasma samples did not show a clade specific inhibitory effect, rather viruses from both the subtype-B and C got affected. This finding is interesting because the plasma antibodies were competed by the same MPER peptide, which implicates the conservation of cross clade binding targets within the MPER, as have been previously appreciated [22], [39], [78], [79]. It is also noteworthy that three of the viruses affected by two competed plasma were common, while others were different, suggesting that the specificities of MPER directed antibodies in these two plasma samples would be possibly similar with some differences. However, the mapping of the IgG fraction from CNP with con-C and B MPER overlapping peptides did not show any reactivity for AIIMS239, possibly due to its very low antibody titers. Nonetheless, the CNP AIIMS206 showed binding to peptides containing 2F5 epitope ELDKWA in con-B MPER and also reacted to its con-C counterpart to which mAb 2F5 failed to bind, since it lacks a complete 2F5 epitope. Taken together, we observed MPER reactive antibodies in the CNP AIIMS206 whose epitopes overlap with mAb 2F5, however displays slightly different specificity. Indeed, a number of groups have recently shown that anti-MPER antibodies are found in samples with neutralization breadth that in some cases were identified as 2F5-like [79], or 4E10-like, Z13-like [7], [80]. Moreover, we were unable to completely remove the neutralizing activity from any of the three CNP sample, which suggests that MPER may not solely contribute towards the viral neutralization, instead the possibility that antibodies specific for oligomeric forms of envelope (quaternary epitopes) may define this activity [81]–[85].

Overall, our data reveals that broadly cross-neutralizing Abs can be detected in approximately 19–27% of the plasma samples derived from subtype-C HIV-1 infected Indian patients. The neutralization profiles of the plasma will allow the identification of HIV-1 donors for the production of neutralizing mAbs and also will help to guide the design of new vaccine immunogens. We explore here for the first time the antigencity of major immunogenic sites on HIV-1 infecting Indian patients and our results suggest the importance of MPER directed antibodies for HIV-1 neutralization.

## Supporting Information

Figure S1 Relative reactivity of plasma antibodies form HIV-1 infected individuals against V3, MPER and IDR peptides. Relative anti-V3, anti-MPER and anti-IDR antibody titers in 80 HIV-1 infected drug naive patients. The plasma were reacted with V3 loop (35 mer), MPER (24 mer) and IDR (19 mer) peptides at six dilutions (dilution range: 300 to 100000) in an ELISA binding assay. The colour bars represent the reciprocal 50% binding (Max50) titers against V3 (red), MPER (green) and IDR (blue) regions. The Max50 binding titers were calculated by least square regression method using Graphpad Prism 5.
(TIF)

Figure S2 Association of Neutralization by plasma antibodies with Max50 ELISA binding titers to major antigenic regions on HIV-1 envelope. The reciprocal mean ID50 neutralization titers of all the tested viruses (black), subtype-C (gold) and subtype-B (aqua) viruses and were compared by spearman rank correlation with Max50 ELISA binding titers to third variable region (V3: red), membrane proximal external region (MPER: green) and immunodominant region (IDR: blue) of envelope glycoprotein gp160. Also the same statistical test was used to compare the Max50 binding values and mean neutralization titers of individual viral isolates. The analysis was done with 80 HIV-1 plasma samples and the p-values are given for each category.
(TIF)

Table S1 Demographic and clinical data of 80 HIV-1 infected drug naive patients recruited for the study.
(DOC)

Table S2 The neutralizing activity of broadly neutralizing antibodies against viruses tested in this study. The neutralizing activity of broadly neutralizing monoclonal antibodies bNAbs (indicated on the top) was assessed against the six reference subtype_B and C and two new subtype_C viruses (left), using the TZM-bl cell assay. The mAb 1418 specific to parvovirus B19 protein was used as negative control in neutralization assay. The numerical values below the mAbs represent IC50 neutralization titers (which is the amount of mAbs (µg/ml) needed for 50% neutralization) against each virus. The IC50 values which are shown in each cell are in coded: IC50<1 µg/ml (Bold); IC50, 1–30 µg/ml (Italic); IC50>30 indicates that IC50 was not achieved. The two new isolates (AIIMS201 and AIIMS212) showed resistance to neutralization by bNAbs and were assigned as tier 2 viruses in this study.
(DOC)

Table S3 **A**. Epitope mapping of polyclonal antibodies from cross-neutralizing plasma (CNP) with overlapping linear peptides corresponding to HIV-1 consensus-C gp120. The polyclonal antibodies from three CNPs (AIIMS206, AIIMS239 and AIIMS249) and two seronegative healthy donors (A1 and A2) were reacted with 15 mer linear overlapping peptides (11 amino acid overlap) corresponding to the HIV-1 consensus-C gp120 amino acid sequence (sequences for each peptide are provided on the left of table), at four dilutions (dilution range: 100 to 3000) in an ELISA binding assay. The numerical values in boxes are the reciprocal Max50 binding titers, calculated by Graphpad Prism 5 using least square regression method. The information is coded: (Bold) Max50>1000, (Italic) Max50 = 101–1000 and unfilled Max50<100 indicates the Max50 was not achieved. **B**. Epitope mapping of polyclonal antibodies from cross-neutralizing plasma (CNP) with overlapping linear peptides corresponding to HIV-1 consensus-C gp41. The polyclonal antibodies from three CNPs (AIIMS206, AIIMS239 and AIIMS249) and two seronegative healthy donors (A1 and A2) were reacted with 15 mer linear overlapping peptides (11 amino acid overlap) corresponding to the HIV-1 consensus-C gp41 amino acid sequence (sequences for each peptide are provided on the left of table), at four dilutions (dilution range: 100 to 3000) in an ELISA binding assay. The numerical values in boxes are the reciprocal Max50 binding titers, calculated by Graphpad Prism 5 using least square regression method. The information is coded: (Bold) Max50>1000, (Italic) Max50 = 101–1000 and unfilled Max50<100 indicates the Max50 was not achieved.
(DOC)
