# Supplementary figures and images for: Neutralization of Tier-2 Viruses and Epitope Profiling of Plasma Antibodies from Human Immunodeficiency Virus Type 1 Infected Donors from India
Source: PLoS One. 2012 Aug 31;7(8):e43704. doi: 10.1371/journal.pone.0043704 (PMC3432049; doi:10.1371/journal.pone.0043704)

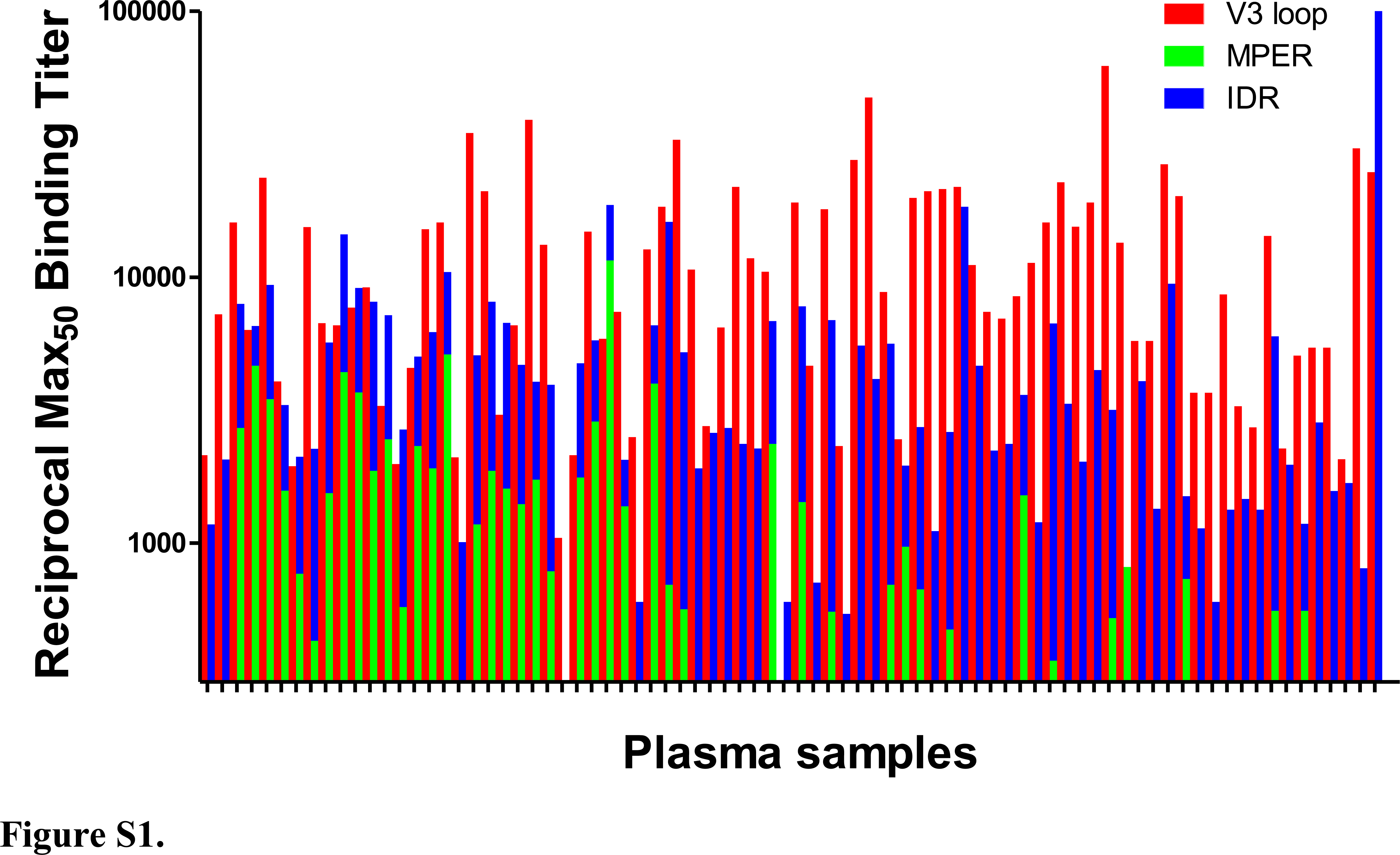

Supplement: Figure S1 — Relative reactivity of plasma antibodies form HIV-1 infected individuals against V3, MPER and IDR peptides. Relative anti-V3, anti-MPER and anti-IDR antibody titers in 80 HIV-1 infected drug naive patients. The plasma were reacted with V3 loop (35 mer), MPER (24 mer) and IDR (19 mer) peptides at six dilutions (dilution range: 300 to 100000) in an ELISA binding assay. The colour bars represent the reciprocal 50% binding (Max50) titers against V3 (red), MPER (green) and IDR (blue) regions. The Max50 binding titers were calculated by least square regression method using Graphpad Prism 5. (TIF) [file pone.0043704.s001.tif]

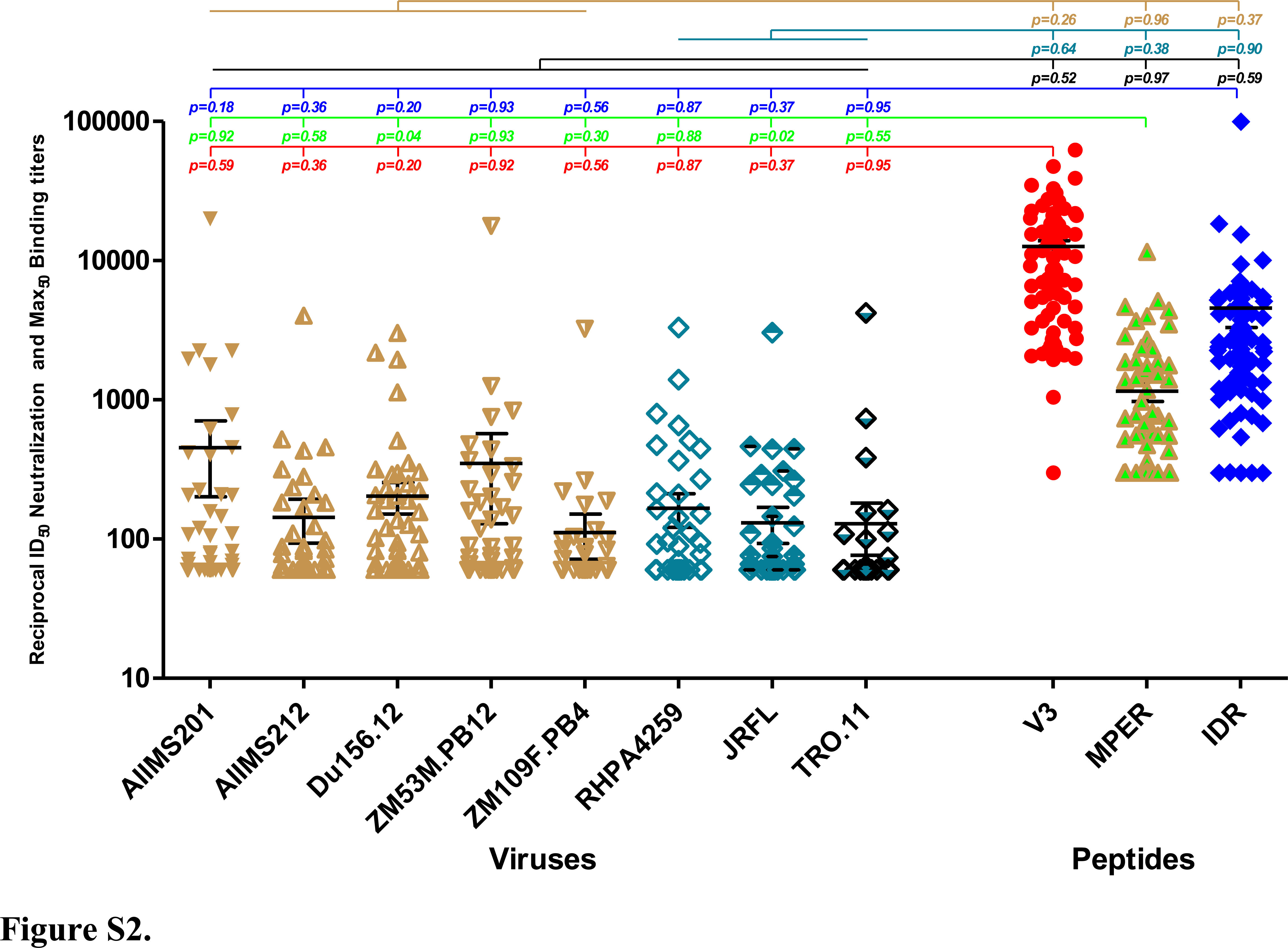

Supplement: Figure S2 — Association of Neutralization by plasma antibodies with Max50 ELISA binding titers to major antigenic regions on HIV-1 envelope. The reciprocal mean ID50 neutralization titers of all the tested viruses (black), subtype-C (gold) and subtype-B (aqua) viruses and were compared by spearman rank correlation with Max50 ELISA binding titers to third variable region (V3: red), membrane proximal external region (MPER: green) and immunodominant region (IDR: blue) of envelope glycoprotein gp160. Also the same statistical test was used to compare the Max50 binding values and mean neutralization titers of individual viral isolates. The analysis was done with 80 HIV-1 plasma samples and the p-values are given for each category. (TIF) [file pone.0043704.s002.tif]
