# Supplementary material for: Neutralization of Tier-2 Viruses and Epitope Profiling of Plasma Antibodies from Human Immunodeficiency Virus Type 1 Infected Donors from India
Source: PLoS One. 2012 Aug 31;7(8):e43704. doi: 10.1371/journal.pone.0043704 (PMC3432049; doi:10.1371/journal.pone.0043704)
Supplement: Table S1 — Demographic and clinical data of 80 HIV-1 infected drug naive patients recruited for the study. (DOC) [file pone.0043704.s003.doc]

**Table S1. Demographic and clinical data of 80 HIV-1 infected drug naive patients recruited for the study**

| **Parameters** | **Values** |
| --- | --- |
| Age (years), median (range) | 32 (20-57) |
| Gender (M/F) | 50/30 |
| CD4 count (per cubic millimetre), median (range) | 337.5 (14-966) |
| Plasma viral load (RNA copies/ml), median (range) | 30800 (47-2180000)**^1^** |
| Plasma IgG (mg/ml), (mean±SD) | 12.28 ±0.45**^2^** |
| Time since first diagnosis (days), median (range) | 235.5 (2-2555) |

^1^ Viral load data is shown for 53 patient samples.

^2^ Plasma total IgG levels were determined for 65 patient samples.
