# Supplementary material for: Neutralization of Tier-2 Viruses and Epitope Profiling of Plasma Antibodies from Human Immunodeficiency Virus Type 1 Infected Donors from India
Source: PLoS One. 2012 Aug 31;7(8):e43704. doi: 10.1371/journal.pone.0043704 (PMC3432049; doi:10.1371/journal.pone.0043704)
Supplement: Table S2 — The neutralizing activity of broadly neutralizing antibodies against viruses tested in this study. The neutralizing activity of broadly neutralizing monoclonal antibodies bNAbs (indicated on the top) was assessed against the six reference subtype_B and C and two new subtype_C viruses (left), using the TZM-bl cell assay. The mAb 1418 specific to parvovirus B19 protein was used as negative control in neutralization assay. The numerical values below the mAbs represent IC50 neutralization titers (which is the amount of mAbs (µg/ml) needed for 50% neutralization) against each virus. The IC50 values which are shown in each cell are in coded: IC50<1 µg/ml (Bold); IC50, 1–30 µg/ml (Italic); IC50>30 indicates that IC50 was not achieved. The two new isolates (AIIMS201 and AIIMS212) showed resistance to neutralization by bNAbs and were assigned as tier 2 viruses in this study. (DOC) [file pone.0043704.s004.doc]

Table S2. The neutralizing activity of broadly neutralizing antibodies against viruses tested in this study.

|  |  |  | **mAbs** | | | | | |
| --- | --- | --- | --- | --- | --- | --- | --- | --- |
| **#** | **Virus** | **clade** | **2F5** | **4E10** | **b12** | **2G12** | **447** | **1418** |
| 1 | JRFL | B | *1.2* | *15* | **<0.1** | **0.2** | 20 | >30 |
| 2 | TRO.11 | B | >30 | **<0.1** | >30 | **0.3** | >30 | >30 |
| 3 | RHPA4259.7 | B | *17* | *3.5* | **<0.1** | >30 | >30 | >30 |
| 4 | ZM109F.PB4 | C | >30 | *3* | *27* | >30 | >30 | >30 |
| 5 | Du156.12 | C | >30 | **<0.1** | **<0.1** | >30 | >30 | >30 |
| 6 | ZM53M.PB12 | C | >30 | *4* | >30 | >30 | >30 | >30 |
| 7 | AIIMS201 | C | >30 | >30 | >30 | >30 | >30 | >30 |
| 8 | AIIMS212 | C | >30 | >30 | >30 | >30 | >30 | >30 |
