# Supplementary material for: Neutralization of Tier-2 Viruses and Epitope Profiling of Plasma Antibodies from Human Immunodeficiency Virus Type 1 Infected Donors from India
Source: PLoS One. 2012 Aug 31;7(8):e43704. doi: 10.1371/journal.pone.0043704 (PMC3432049; doi:10.1371/journal.pone.0043704)
Supplement: Table S3 — A. Epitope mapping of polyclonal antibodies from cross-neutralizing plasma (CNP) with overlapping linear peptides corresponding to HIV-1 consensus-C gp120. The polyclonal antibodies from three CNPs (AIIMS206, AIIMS239 and AIIMS249) and two seronegative healthy donors (A1 and A2) were reacted with 15 mer linear overlapping peptides (11 amino acid overlap) corresponding to the HIV-1 consensus-C gp120 amino acid sequence (sequences for each peptide are provided on the left of table), at four dilutions (dilution range: 100 to 3000) in an ELISA binding assay. The numerical values in boxes are the reciprocal Max50 binding titers, calculated by Graphpad Prism 5 using least square regression method. The information is coded: (Bold) Max50>1000, (Italic) Max50 = 101–1000 and unfilled Max50<100 indicates the Max50 was not achieved. B. Epitope mapping of polyclonal antibodies from cross-neutralizing plasma (CNP) with overlapping linear peptides corresponding to HIV-1 consensus-C gp41. The polyclonal antibodies from three CNPs (AIIMS206, AIIMS239 and AIIMS249) and two seronegative healthy donors (A1 and A2) were reacted with 15 mer linear overlapping peptides (11 amino acid overlap) corresponding to the HIV-1 consensus-C gp41 amino acid sequence (sequences for each peptide are provided on the left of table), at four dilutions (dilution range: 100 to 3000) in an ELISA binding assay. The numerical values in boxes are the reciprocal Max50 binding titers, calculated by Graphpad Prism 5 using least square regression method. The information is coded: (Bold) Max50>1000, (Italic) Max50 = 101–1000 and unfilled Max50<100 indicates the Max50 was not achieved. (DOC) [file pone.0043704.s005.doc]

**Table S3a. Epitope mapping of polyclonal antibodies from cross-neutralizing plasma (CNP) with overlapping linear peptides corresponding to HIV-1 consensus-C gp120.**

| **Cat No** | **Sequence** | **AIIMS206** | **AIIMS239** | **AIIMS249** | **A1** | **A2** |
| --- | --- | --- | --- | --- | --- | --- |
| **9185** | MRVRGILRNCQQWWI | <100 | <100 | <100 | <100 | <100 |
| **9186** | GILRNCQQWWIWGIL | <100 | <100 | <100 | <100 | <100 |
| **9187** | NCQQWWIWGILGFWM | <100 | <100 | <100 | <100 | <100 |
| **9188** | WWIWGILGFWMLMIC | <100 | <100 | <100 | <100 | <100 |
| **9189** | GILGFWMLMICNVMG | <100 | <100 | <100 | <100 | <100 |
| **9190** | FWMLMICNVMGNLWV | <100 | <100 | <100 | <100 | <100 |
| **9191** | MICNVMGNLWVTVYY | <100 | <100 | <100 | <100 | <100 |
| **9192** | VMGNLWVTVYYGVPV | <100 | <100 | <100 | <100 | <100 |
| **9193** | LWVTVYYGVPVWKEA | <100 | <100 | <100 | <100 | <100 |
| **9194** | VYYGVPVWKEAKTTL | <100 | <100 | <100 | <100 | <100 |
| **9195** | VPVWKEAKTTLFCAS | <100 | <100 | <100 | <100 | <100 |
| **9196** | KEAKTTLFCASDAKA | <100 | <100 | <100 | <100 | <100 |
| **9197** | TTLFCASDAKAYETE | <100 | <100 | <100 | <100 | <100 |
| **9198** | CASDAKAYETEVHNV | <100 | <100 | <100 | <100 | <100 |
| **9199** | AKAYETEVHNVWATH | <100 | <100 | <100 | <100 | <100 |
| **9200** | ETEVHNVWATHACVP | <100 | <100 | <100 | <100 | <100 |
| **9201** | HNVWATHACVPTDPN | <100 | <100 | <100 | <100 | <100 |
| **9202** | ATHACVPTDPNPQEM | <100 | <100 | <100 | <100 | <100 |
| **9203** | CVPTDPNPQEMVLEN | <100 | <100 | <100 | <100 | <100 |
| **9204** | DPNPQEMVLENVTEN | <100 | <100 | <100 | <100 | <100 |
| **9205** | QEMVLENVTENFNMW | <100 | <100 | <100 | <100 | <100 |
| **9206** | LENVTENFNMWKNDM | <100 | <100 | <100 | <100 | <100 |
| **9207** | TENFNMWKNDMVDQM | <100 | <100 | <100 | <100 | <100 |
| **9208** | NMWKNDMVDQMHEDI | <100 | <100 | <100 | <100 | <100 |
| **9209** | NDMVDQMHEDIISLW | <100 | <100 | <100 | <100 | <100 |
| **9210** | DQMHEDIISLWDQSL | <100 | <100 | <100 | <100 | <100 |
| **9211** | EDIISLWDQSLKPCV | <100 | <100 | <100 | <100 | <100 |
| **9212** | SLWDQSLKPCVKLTP | <100 | <100 | <100 | <100 | <100 |
| **9213** | QSLKPCVKLTPLCVT | <100 | <100 | <100 | <100 | <100 |
| **9214** | PCVKLTPLCVTLNCR | <100 | <100 | <100 | <100 | <100 |
| **9215** | LTPLCVTLNCRNNVT | <100 | <100 | <100 | <100 | <100 |
| **9216** | CVTLNCRNNVTNNAT | <100 | <100 | <100 | <100 | <100 |
| **9217** | NCRNNVTNNATNNNN | <100 | <100 | <100 | <100 | <100 |
| **9218** | NVTNNATNNNNNTMK | <100 | <100 | <100 | <100 | <100 |
| **9219** | NATNNNNNTMKEEIK | <100 | <100 | <100 | <100 | <100 |
| **9220** | NNNNTMKEEIKNCSF | <100 | <100 | <100 | <100 | <100 |
| **9221** | TMKEEIKNCSFNITT | <100 | <100 | <100 | <100 | <100 |
| **9222** | EIKNCSFNITTELRD | <100 | <100 | <100 | <100 | <100 |
| **9223** | CSFNITTELRDKKQK | <100 | <100 | <100 | <100 | <100 |
| **9224** | ITTELRDKKQKVYAL | <100 | <100 | <100 | <100 | <100 |
| **9225** | LRDKKQKVYALFYRL | *109* | <100 | <100 | <100 | *151* |
| **9226** | KQKVYALFYRLDIVP | <100 | <100 | <100 | <100 | <100 |
| **9227** | YALFYRLDIVPLNEK | <100 | <100 | <100 | <100 | <100 |
| **9228** | YRLDIVPLNEKNNSN | <100 | <100 | <100 | <100 | <100 |
| **9229** | IVPLNEKNNSNYSSD | <100 | <100 | <100 | <100 | <100 |
| **9230** | NEKNNSNYSSDYRLI | <100 | <100 | <100 | <100 | <100 |
| **9231** | NSNYSSDYRLINCNT | <100 | <100 | <100 | <100 | <100 |
| **9232** | SSDYRLINCNTSAIT | <100 | <100 | <100 | <100 | <100 |
| **9233** | RLINCNTSAITQACP | *113* | <100 | <100 | <100 | <100 |
| **9234** | CNTSAITQACPKVSF | <100 | <100 | <100 | <100 | <100 |
| **9235** | AITQACPKVSFDPIP | <100 | <100 | <100 | <100 | <100 |
| **9236** | ACPKVSFDPIPIHYC | <100 | <100 | <100 | <100 | <100 |
| **9237** | VSFDPIPIHYCAPAG | <100 | <100 | <100 | <100 | <100 |
| **9238** | PIPIHYCAPAGYAIL | <100 | <100 | <100 | <100 | <100 |
| **9239** | HYCAPAGYAILKCNN | <100 | <100 | *121* | *774* | <100 |
| **9240** | PAGYAILKCNNKTFN | <100 | <100 | <100 | <100 | <100 |
| **9241** | AILKCNNKTFNGTGP | <100 | <100 | <100 | <100 | <100 |
| **9242** | CNNKTFNGTGPCNNV | <100 | <100 | <100 | <100 | <100 |
| **9243** | TFNGTGPCNNVSTVQ | <100 | <100 | <100 | <100 | <100 |
| **9244** | TGPCNNVSTVQCTHG | <100 | <100 | <100 | <100 | <100 |
| **9245** | NNVSTVQCTHGIKPV | <100 | <100 | <100 | <100 | <100 |
| **9246** | TVQCTHGIKPVVSTQ | <100 | <100 | <100 | <100 | <100 |
| **9247** | THGIKPVVSTQLLLN | <100 | <100 | <100 | <100 | <100 |
| **9248** | KPVVSTQLLLNGSLA | <100 | <100 | <100 | <100 | <100 |
| **9249** | STQLLLNGSLAEEEI | <100 | <100 | <100 | <100 | <100 |
| **9250** | LLNGSLAEEEIIIRS | <100 | <100 | <100 | <100 | <100 |
| **9251** | SLAEEEIIIRSENLT | <100 | <100 | <100 | <100 | <100 |
| **9252** | EEIIIRSENLTNNAK | <100 | <100 | <100 | <100 | <100 |
| **9253** | IRSENLTNNAKTIIV | <100 | <100 | <100 | <100 | <100 |
| **9254** | NLTNNAKTIIVHLNE | <100 | <100 | <100 | <100 | <100 |
| **9255** | NAKTIIVHLNESVEI | <100 | <100 | <100 | <100 | <100 |
| **9256** | IIVHLNESVEIVCTR | <100 | <100 | <100 | <100 | <100 |
| **9257** | LNESVEIVCTRPNNN | <100 | <100 | <100 | <100 | <100 |
| **9258** | VEIVCTRPNNNTRKS | <100 | <100 | <100 | <100 | <100 |
| **9259** | CTRPNNNTRKSIRIG | *406* | *227* | <100 | <100 | <100 |
| **9260** | NNNTRKSIRIGPGQT | **>3000** | <100 | <100 | <100 | <100 |
| **9261** | RKSIRIGPGQTFYAT | **1939** | *150* | *227* | <100 | <100 |
| **9262** | RIGPGQTFYATGDII | <100 | <100 | <100 | <100 | <100 |
| **9263** | GQTFYATGDIIGDIR | <100 | <100 | <100 | <100 | <100 |
| **9264** | YATGDIIGDIRQAHC | <100 | <100 | <100 | <100 | <100 |
| **9265** | DIIGDIRQAHCNISE | <100 | <100 | <100 | <100 | <100 |
| **9266** | DIRQAHCNISEEKWN | <100 | <100 | <100 | <100 | <100 |
| **9267** | AHCNISEEKWNKTLQ | <100 | <100 | <100 | <100 | <100 |
| **9268** | ISEEKWNKTLQRVSE | <100 | <100 | <100 | <100 | <100 |
| **9269** | KWNKTLQRVSEKLKE | <100 | <100 | <100 | <100 | <100 |
| **9270** | TLQRVSEKLKEHFPN | <100 | <100 | <100 | <100 | <100 |
| **9271** | VSEKLKEHFPNKTIK | <100 | <100 | <100 | <100 | <100 |
| **9272** | LKEHFPNKTIKFAPS | <100 | <100 | <100 | <100 | <100 |
| **9273** | FPNKTIKFAPSSGGD | <100 | <100 | <100 | <100 | <100 |
| **9274** | TIKFAPSSGGDLEIT | <100 | <100 | <100 | <100 | <100 |
| **9275** | APSSGGDLEITTHSF | <100 | <100 | <100 | <100 | <100 |
| **9276** | GGDLEITTHSFNCRG | <100 | <100 | <100 | <100 | <100 |
| **9277** | EITTHSFNCRGEFFY | <100 | <100 | <100 | <100 | <100 |
| **9278** | HSFNCRGEFFYCNTS | <100 | <100 | <100 | <100 | <100 |
| **9279** | CRGEFFYCNTSKLFN | <100 | <100 | <100 | <100 | <100 |
| **9280** | FFYCNTSKLFNSTYM | <100 | <100 | <100 | <100 | <100 |
| **9281** | NTSKLFNSTYMPNST | <100 | <100 | <100 | <100 | <100 |
| **9282** | LFNSTYMPNSTNNTN | <100 | <100 | <100 | <100 | <100 |
| **9283** | TYMPNSTNNTNTTIT | <100 | <100 | <100 | <100 | <100 |
| **9284** | NSTNNTNTTITLPCR | <100 | <100 | <100 | <100 | <100 |
| **9285** | NTNTTITLPCRIKQI | <100 | <100 | <100 | <100 | <100 |
| **9286** | TITLPCRIKQIINMW | <100 | <100 | <100 | <100 | <100 |
| **9287** | PCRIKQIINMWQEVG | <100 | <100 | <100 | <100 | <100 |
| **9288** | KQIINMWQEVGRAMY | <100 | <100 | <100 | <100 | <100 |
| **9289** | NMWQEVGRAMYAPPI | <100 | <100 | <100 | <100 | <100 |
| **9290** | EVGRAMYAPPIEGNI | <100 | <100 | <100 | <100 | <100 |
| **9291** | AMYAPPIEGNITCKS | <100 | <100 | <100 | <100 | <100 |
| **9292** | PPIEGNITCKSNITG | <100 | <100 | <100 | <100 | <100 |
| **9293** | GNITCKSNITGLLLT | <100 | <100 | <100 | <100 | <100 |
| **9294** | CKSNITGLLLTRDGG | <100 | <100 | <100 | <100 | <100 |
| **9295** | ITGLLLTRDGGKNDT | <100 | <100 | <100 | <100 | <100 |
| **9296** | LLTRDGGKNDTNDTE | *123* | <100 | <100 | <100 | <100 |
| **9297** | DGGKNDTNDTETFRP | <100 | <100 | <100 | <100 | <100 |
| **9298** | NDTNDTETFRPGGGD | <100 | <100 | <100 | <100 | <100 |
| **9299** | DTETFRPGGGDMRDN | <100 | <100 | <100 | <100 | <100 |
| **9300** | FRPGGGDMRDNWRSE | <100 | <100 | <100 | <100 | <100 |
| **9301** | GGDMRDNWRSELYKY | <100 | <100 | <100 | <100 | <100 |
| **9302** | RDNWRSELYKYKVVE | <100 | <100 | <100 | <100 | <100 |
| **9303** | RSELYKYKVVEIKPL | <100 | <100 | <100 | <100 | <100 |
| **9304** | YKYKVVEIKPLGVAP | <100 | <100 | <100 | <100 | <100 |
| **9305** | VVEIKPLGVAPTKAK | <100 | <100 | <100 | <100 | <100 |
| **9306** | KPLGVAPTKAKRRVV | *215* | <100 | <100 | <100 | <100 |
| **9307** | VAPTKAKRRVVEREK | *181* | <100 | <100 | <100 | <100 |

**Table S3b. Epitope mapping of polyclonal antibodies from cross-neutralizing plasma (CNP) with overlapping linear peptides corresponding to HIV-1 consensus-C gp41.**

| **Cat No** | **Sequence** | **AIIMS206** | **AIIMS239** | **AIIMS249** | **A1** | **A2** |
| --- | --- | --- | --- | --- | --- | --- |
| **9308** | KAKRRVVEREKRAVG | *199* | <100 | <100 | <100 | <100 |
| **9309** | RVVEREKRAVGIGAV | *109* | <100 | <100 | <100 | <100 |
| **9310** | REKRAVGIGAVFLGF | <100 | <100 | <100 | <100 | <100 |
| **9311** | AVGIGAVFLGFLGAA | <100 | <100 | <100 | <100 | <100 |
| **9312** | GAVFLGFLGAAGSTM | <100 | <100 | <100 | <100 | <100 |
| **9313** | LGFLGAAGSTMGAAS | <100 | <100 | <100 | <100 | <100 |
| **9314** | GAAGSTMGAASITLT | <100 | <100 | <100 | <100 | <100 |
| **9315** | STMGAASITLTVQAR | <100 | <100 | <100 | <100 | <100 |
| **9316** | AASITLTVQARQLLS | <100 | <100 | <100 | <100 | <100 |
| **9317** | TLTVQARQLLSGIVQ | <100 | <100 | <100 | <100 | <100 |
| **9318** | QARQLLSGIVQQQSN | <100 | <100 | <100 | <100 | <100 |
| **9319** | LLSGIVQQQSNLLRA | <100 | <100 | <100 | <100 | <100 |
| **9320** | IVQQQSNLLRAIEAQ | <100 | <100 | <100 | <100 | <100 |
| **9321** | QSNLLRAIEAQQHML | <100 | <100 | <100 | <100 | <100 |
| **9322** | LRAIEAQQHMLQLTV | <100 | <100 | <100 | <100 | <100 |
| **9323** | EAQQHMLQLTVWGIK | <100 | <100 | <100 | <100 | <100 |
| **9324** | HMLQLTVWGIKQLQT | <100 | <100 | <100 | <100 | <100 |
| **9325** | LTVWGIKQLQTRVLA | <100 | <100 | <100 | <100 | <100 |
| **9326** | GIKQLQTRVLAIERY | <100 | <100 | <100 | <100 | <100 |
| **9327** | LQTRVLAIERYLKDQ | <100 | <100 | <100 | <100 | <100 |
| **9328** | VLAIERYLKDQQLLG | <100 | <100 | <100 | <100 | <100 |
| **9329** | ERYLKDQQLLGIWGC | **>3000** | *126* | *168* | <100 | <100 |
| **9330** | KDQQLLGIWGCSGKL | **1477** | <100 | *113* | <100 | <100 |
| **9331** | LLGIWGCSGKLICTT | **>3000** | *103* | *102* | <100 | <100 |
| **9332** | WGCSGKLICTTAVPW | **>3000** | *117* | *175* | <100 | <100 |
| **9333** | GKLICTTAVPWNSSW | **1834** | <100 | <100 | <100 | <100 |
| **9334** | CTTAVPWNSSWSNKS | <100 | <100 | <100 | <100 | <100 |
| **9335** | VPWNSSWSNKSQEDI | <100 | <100 | <100 | <100 | <100 |
| **9336** | SSWSNKSQEDIWDNM | <100 | <100 | <100 | <100 | <100 |
| **9337** | NKSQEDIWDNMTWMQ | <100 | <100 | <100 | <100 | <100 |
| **9338** | EDIWDNMTWMQWDRE | <100 | <100 | <100 | <100 | <100 |
| **9339** | DNMTWMQWDREISNY | *246* | <100 | <100 | <100 | <100 |
| **9340** | WMQWDREISNYTDTI | <100 | <100 | <100 | <100 | <100 |
| **9341** | DREISNYTDTIYRLL | <100 | <100 | <100 | <100 | <100 |
| **9342** | SNYTDTIYRLLEDSQ | <100 | <100 | <100 | <100 | <100 |
| **9343** | DTIYRLLEDSQNQQE | <100 | <100 | <100 | <100 | <100 |
| **9344** | RLLEDSQNQQEKNEK | <100 | <100 | <100 | <100 | <100 |
| **9345** | DSQNQQEKNEKDLLA | <100 | <100 | <100 | <100 | <100 |
| **9346** | QQEKNEKDLLALDSW | *112* | <100 | <100 | <100 | <100 |
| **9347** | NEKDLLALDSWKNLW | <100 | <100 | <100 | <100 | <100 |
| **9348** | LLALDSWKNLWNWFD | <100 | <100 | <100 | <100 | <100 |
| **9349** | DSWKNLWNWFDITNW | <100 | <100 | <100 | <100 | <100 |
| **9350** | NLWNWFDITNWLWYI | <100 | <100 | <100 | <100 | <100 |
| **9351** | WFDITNWLWYIKIFI | <100 | <100 | <100 | <100 | <100 |
| **9352** | TNWLWYIKIFIMIVG | *122* | <100 | <100 | <100 | <100 |
| **9353** | WYIKIFIMIVGGLIG | <100 | <100 | <100 | <100 | <100 |
| **9354** | IFIMIVGGLIGLRII | <100 | <100 | <100 | <100 | <100 |
| **9355** | IVGGLIGLRIIFAVL | <100 | <100 | <100 | <100 | <100 |
| **9356** | LIGLRIIFAVLSIVN | <100 | <100 | <100 | <100 | <100 |
| **9357** | RIIFAVLSIVNRVRQ | <100 | <100 | <100 | <100 | <100 |
| **9358** | AVLSIVNRVRQGYSP | <100 | <100 | <100 | <100 | <100 |
| **9359** | IVNRVRQGYSPLSFQ | <100 | <100 | <100 | <100 | <100 |
| **9360** | VRQGYSPLSFQTLTP | <100 | <100 | <100 | <100 | <100 |
| **9361** | YSPLSFQTLTPNPRG | <100 | <100 | <100 | <100 | <100 |
| **9362** | SFQTLTPNPRGPDRL | <100 | <100 | <100 | <100 | <100 |
| **9363** | LTPNPRGPDRLGRIE | <100 | <100 | <100 | <100 | <100 |
| **9364** | PRGPDRLGRIEEEGG | <100 | <100 | <100 | <100 | <100 |
| **9365** | DRLGRIEEEGGEQDR | <100 | <100 | <100 | <100 | <100 |
| **9366** | RIEEEGGEQDRDRSI | <100 | <100 | <100 | <100 | <100 |
| **9367** | EGGEQDRDRSIRLVN | <100 | <100 | <100 | <100 | <100 |
| **9368** | QDRDRSIRLVNGFLA | <100 | <100 | <100 | <100 | <100 |
| **9369** | RSIRLVNGFLALAWD | <100 | <100 | <100 | <100 | <100 |
| **9370** | LVNGFLALAWDDLRS | <100 | <100 | <100 | <100 | <100 |
| **9371** | FLALAWDDLRSLCLF | <100 | <100 | <100 | <100 | <100 |
| **9372** | AWDDLRSLCLFSYHR | <100 | <100 | <100 | <100 | <100 |
| **9373** | LRSLCLFSYHRLRDF | <100 | <100 | <100 | <100 | <100 |
| **9374** | CLFSYHRLRDFILIA | <100 | <100 | <100 | <100 | <100 |
| **9375** | YHRLRDFILIAARAV | <100 | <100 | <100 | <100 | <100 |
| **9376** | RDFILIAARAVELLG | <100 | <100 | <100 | <100 | <100 |
| **9377** | LIAARAVELLGRSSL | <100 | <100 | <100 | <100 | <100 |
| **9378** | RAVELLGRSSLRGLQ | <100 | <100 | <100 | <100 | <100 |
| **9379** | LLGRSSLRGLQRGWE | <100 | <100 | <100 | <100 | <100 |
| **9380** | SSLRGLQRGWEALKY | <100 | <100 | <100 | <100 | <100 |
| **9381** | GLQRGWEALKYLGSL | <100 | <100 | <100 | <100 | <100 |
| **9382** | GWEALKYLGSLVQYW | <100 | <100 | <100 | <100 | <100 |
| **9383** | LKYLGSLVQYWGLEL | <100 | <100 | <100 | <100 | <100 |
| **9384** | GSLVQYWGLELKKSA | <100 | <100 | <100 | <100 | <100 |
| **9385** | QYWGLELKKSAISLL | <100 | <100 | <100 | <100 | <100 |
| **9386** | LELKKSAISLLDTIA | <100 | <100 | <100 | <100 | <100 |
| **9387** | KSAISLLDTIAIAVA | <100 | <100 | <100 | <100 | <100 |
| **9388** | SLLDTIAIAVAEGTD | <100 | <100 | <100 | <100 | <100 |
| **9389** | TIAIAVAEGTDRIIE | <100 | <100 | <100 | <100 | <100 |
| **9390** | AVAEGTDRIIELIQR | <100 | <100 | <100 | <100 | <100 |
| **9391** | GTDRIIELIQRICRA | <100 | <100 | <100 | <100 | <100 |
| **9392** | IIELIQRICRAIRNI | <100 | <100 | <100 | <100 | <100 |
| **9393** | IQRICRAIRNIPRRI | <100 | <100 | <100 | <100 | <100 |
| **9394** | CRAIRNIPRRIRQGF | *222* | <100 | <100 | *113* | *156* |
| **9395** | RNIPRRIRQGFEAAL | <100 | <100 | <100 | <100 | <100 |
| **9396** | RRIRQGFEAALQ | <100 | <100 | <100 | <100 | <100 |
